# Impact of Gene–Environment Interactions on Cancer Development

**DOI:** 10.3390/ijerph17218089

**Published:** 2020-11-03

**Authors:** Ariane Mbemi, Sunali Khanna, Sylvianne Njiki, Clement G. Yedjou, Paul B. Tchounwou

**Affiliations:** 1 NIH/NIMHD RCMI-Center for Health Disparities Research, Jackson State University, 1400 Lynch Street, Box 18750, Jackson, MS 39217, USA; ariane.t.mbemi@jsums.edu (A.M.); sylvianne.njiki@students.jsums.edu (S.N.); 2 Department of Biology, College of Science, Engineering and Technology, Jackson State University, 1400 Lynch Street, Box 18540, Jackson, MS 39217, USA; 3 Department of Oral Medicine and Radiology, Nair Hospital Dental College, Municipal Corporation of Greater Mumbai, Mumbai 400 008, India; sunalikhanna@gmail.com; 4 Department of Biological Sciences, College of Science and Technology, Florida Agricultural and Mechanical University, 1610 S. Martin Luther King Blvd., Tallahassee, FL 32307, USA; clement.yedjou@famu.edu

**Keywords:** gene–environment interactions, genes, environmental exposures, human cancers

## Abstract

Several epidemiological and experimental studies have demonstrated that many human diseases are not only caused by specific genetic and environmental factors but also by gene–environment interactions. Although it has been widely reported that genetic polymorphisms play a critical role in human susceptibility to cancer and other chronic disease conditions, many single nucleotide polymorphisms (SNPs) are caused by somatic mutations resulting from human exposure to environmental stressors. Scientific evidence suggests that the etiology of many chronic illnesses is caused by the joint effect between genetics and the environment. Research has also pointed out that the interactions of environmental factors with specific allelic variants highly modulate the susceptibility to diseases. Hence, many scientific discoveries on gene–environment interactions have elucidated the impact of their combined effect on the incidence and/or prevalence rate of human diseases. In this review, we provide an overview of the nature of gene–environment interactions, and discuss their role in human cancers, with special emphases on lung, colorectal, bladder, breast, ovarian, and prostate cancers.

## 1. Introduction

Cancer is classified as the second leading cause of death worldwide. One in every six deaths that occurred in 2018 was caused by cancer [1,2]. In the United States (US), about 1,806,590 people are expected to be diagnosed with cancer in 2020, corresponding to about 4950 new cases daily, and approximately 606,520 people are also expected to die of cancer in 2020, equivalent to 1600 deaths per day [3]. The lifetime risk of being diagnosed with an invasive cancer is slightly higher for men (40.1%) than women (38.7%), and the variation in cancer incidence by gender may be due to differences in environmental exposure and endogenous hormones, as well as complex interactions between these factors [4,5].

The existence of cell populations that have lost the ability to communicate, grow, and multiply in a normal way is the most common feature of all types of cancer [6,7]. Cancer is commonly defined as a diverse set of diseases in which cells of the human body gain the capacity to divide and duplicate in an uncontrollable manner [7,8]. Each time a cell divides it must duplicate the entire genome and distribute one copy of each chromosome into each daughter nucleus [9,10]. Cell division is regulated when oncogenes are not expressed, and tumor suppressor genes are expressed in healthy cells due to the fact that key regulators of cell division such as cyclins and cyclin-dependent kinases and other cell cycle checkpoints restrict the process [11]. However, the disruption of cell function pathways initiated by environmental factors could lead to an unregulated proliferation of cells [12,13]. Therefore, cancer begins in a single cell when oncogenes are turned on, and the tumor suppressor genes are turned off, shifting the control of cell division [13].

The development of cancer or carcinogenesis has multiple stages, including initiation, promotion, and progression [14,15]. The initiation stage can be caused by exposure to a carcinogenic substance (arsenic, benzene, dioxin, nickel, etc.) or other environmental risk factors that induce genomic instability or disrupt cellular metabolism. The genomic instability results in dysfunction of nucleotides such as base substitution, base loss, nucleotide deletion, insertion or amplification of a base pair, which can further induce DNA breakage, chromosome remodeling, or translocation, and if the injuries are not fixed, can lead to irreversible cell mutation and continuous growth [16,17]. Ninety percent of solid tumors and 50% of hematopoietic cancers are associated with chromosome aberrations or aneuploidy [18]. The promotion phase of carcinogenesis involves an alteration of cells’ environment that favors the development of neoplastic cells. Hence, normal cells mutate and develop into a clone population. Cancer progression involves the aggressive and metastatic phase of the disease [17].

Several risk factors have been attributed to the development of cancer, including the following: lifestyle choices such as cigarette smoking, heavy alcohol drinking, high intake of red meat and fat, and low intake of fiber [19,20]; indoor and outdoor air pollution, along with exposure to chemical substances and radiation [21,22,23]; as well as inherited genes, such as BRCA1 and BRCA2 [24]. The mortality of cancer has slightly decreased in recent years due to medical improvements in early detection, diagnosis, and screening. Even though there is scientific evidence indicating that incidence and mortality rates of cancer have slightly reduced in recent years, further research is needed to better understand the combined effect of the interaction between various risk factors in the process of cancer initiation, promotion, and progression. There exist scientific gaps related to the lack of full understanding of all the specific pathways and mechanisms of cancer development resulting from the interactions of these factors. Recent investigations have pointed out that gene–environmental interactions represent one of the major risk factors associated with cancer etiology [25].

Scientific studies have indicated that genetic polymorphisms are involved in the activation of several biological processes, such as apoptosis, DNA synthesis and methylation, cell cycle regulation, and transcription [26,27]. Despite such genetic influences, the environment and other socio-economic and demographic factors play a crucial role in modifying many other biological determinants of human health. For example, tobacco use, changes in the reproduction pattern, obesity, urbanization, and aging of the population have been associated with environmental factors responsible for cancer [28]. Therefore, scientists are still trying to find out how genetics and the environment interplay in cancer incidence, prevalence, and treatment outcomes. The cause of cancer in a population can be assigned to one of four categories, including inherited susceptible genes alone; environmental factors alone; the interaction between gene and environment; or spontaneous appearance, where the tumors just arise randomly in any part of the human body [29].

On the other hand, Dr. Judith Stern (Distinguished Professor at University of California, Davis)’s statement that “genetics loads the guns but environment pulls the triggers” has been highly articulated, indicating that disease phenotypes not only result from interactions between different genes within the host but also from the interplay between genes and environment [28]. Consequently, research on gene–environment interactions is crucial in understanding how genetic heterogeneity is influenced by various environmental agents [28,29], giving more enlightenment on the biological nature of cancer, improving the capacity to identify the susceptible gene that interacts with other factors [30,31], helping to single out why some individuals exposed to a specific agent develop cancer and others do not, and predicting the population subgroups that are more susceptible to cancer [32,33].

Scientific evidence from epidemiological studies in immigrant populations highlight the prominent role of the environment in the incidence and prevalence of cancer by geographical location [30]. From population-based twin cohort studies, it has been pointed out that more than 50% of cancer incidence is attributed to environmental factors [34,35]. It has also been estimated that a great number of cancer cases could be preventable if substantial measures are taken and applied towards modifiable environmental factors and gene effect. Therefore, this research aims at highlighting the relationship between genetics and environmental exposure in the pathogenesis and development of human cancers. A special emphasis is placed on elucidating the role of gene–environment interactions in the development of the most common cancers, including lung, colorectal, breast, ovarian, and prostate cancers.

## 2. Methods

This literature review focuses on scientific publications targeting both genes and environment as risk factors associated with cancer development. The publications search was conducted on different electronic databases and governmental official websites, such as MEDLINE, PubMed, and Google scholars, as well as the American Cancer Society (ACS), the Centers for Disease Control and Prevention (CDC), the Environmental Protection Agency (EPA), and the World Health Organization (WHO). The cited literature was also screened for relevance and appropriateness. To completely consolidate the relevant publications, different key terms, such as “gene–environment interactions”, “cancer risk factors”, “gene–environment in cancer“, “gene–environment in treatment of cancer”, and “case study of gene–environment interactions and cancer”, were used for the search. A publication was considered relevant if it examined gene–environment interactions in relation to specific types of human cancer. Scientific information obtained from relevant publications was further organized and structured by cancer type. Hence, we collected and examined peer-reviewed publications that provided invaluable information on the potential impact of gene–environment interactions on cancer initiation, promotion, and/or progression. Papers published between 1981 and 2020 were selected for this study.

## 3. Results and Discussion

### 3.1. Evaluation of Gene–Environment Interactions

Studies assessing gene–environment (G × E) interactions on cancer focus on two scales: multiplicative and additive [36,37]. For the G × E interaction at the multiplicative scale, the researcher estimates the joint effect measured in terms of relative risk, the odds ratio of the genetic and environmental factor. The information generated on the relationship of the two components helps in observing if the combined effect is significantly higher or smaller compared to each individual factor [36]. In other words, the multiplicative scale evaluation is made to assess the relative risk of an environmental factor, such as tobacco, heavy metal, organochloride pesticide, etc., with a gene variation or genetic polymorphism. On the other hand, the assessment that is made at the additive scale looks at risk difference rather than the ratio measurement [36]. Therefore, at the additive scale, G × E interactions assessment examines if an individual with a specific genotype and exposed to a high or low dose of an environmental factor is associated with cancer risk [36,38]. However, it has been pointed out that the sample size needed to investigate G × E interactions with enough scientific power is one of the biggest issues encountered by many investigators [38]. However, as a rule of thumb, published research has recommended that “studies aiming at evaluating departure from multiplicatively have to be at least four times the size needed to investigate effects of the genetic or environmental factor alone” [39].

Mendelian randomization methodologies of the G × E interactions analysis have also been examined. Mendelian randomization utilizes genetic variants that are related to exposures of interest as intermediaries to estimate the exposures. It helps to fortify the potential cause of the modifiable factors [40,41]. Since alleles are randomly assigned during gamete formation and fertilization, the Mendelian randomization methodology on G × E interactions analysis helps to dissociate each allele variant and exposure. For instance, alleles that are associated with coffee consumption should not be associated with lifestyle or demographic factors, which would distort the observational relationship between coffee and cancer progression [41].

A good example is the gene–environment interaction between folate, other B-vitamins, and the polymorphism of genes that are involved in one-carbon metabolism (OCM) influencing the risk of breast cancer [42,43]. A published report demonstrated that folate in the OCM pathway may influence deoxyribonucleic acid (DNA) methylation, nucleotide synthesis, DNA replication and repair, gene expression, and carcinogenesis [44]. In addition, gene mutations in the OCM pathway including methylenetetrahydrofolate reductase (MTHFR) 677 (rs1801133), MTHFR 1298 (rs1801131), and dihydrofolate reductase (DHFR) 19bp (rs70991108) affect the folate-mediated pathway and may result in aberrant methylation and the disruption of DNA synthesis and repair, therefore increasing the risk of breast cancer [43,45]. Furthermore, mutations of these five polymorphism genes of the OCM pathway may affect epigenetic modifications where aberrations such as gene-locus hypermethylation resulting from the silencing of tumor suppressor genes or hypermethylation of certain genes and repetitive sequences may lead to cancer [46,47,48]. Several reports have shown that global hypomethylation increases with age and is also linked to genomic instability and the activation of oncogene expression [49,50,51]. The gene mutations in the OCM pathway affect DNA methylation by disrupting the epigenome, leading to carcinogenesis and inhibition of the normal cellular differentiation processes [52].

Table 1 presents the key findings (expressed in terms of odd ratios, relative risks and/or hazard risks) from various epidemiological studies of gene-environment interactions together with specific gene variants and environmental exposures. 

### 3.2. Gene–Environment Interactions in the Development of Lung Cancer

The public health burden of lung cancer continues to increase worldwide. In the US, lung cancer is the primary cause of cancer deaths [59]. It is viewed as a heterogeneous disease associated mainly with exposure to tobacco. It has been established that tobacco smoking accounts for more than 60% of the risk of lung cancer [60]. Cigarettes contain more than 7000 hazardous chemicals listed as inflammation- and cancer-causing agents in humans [61]. Exposure to cigarette smoke is a cause of lung cancer, which disproportionally affects people living with low-income and/or in poor neighborhoods. It also causes a great negative economic impact on minority and underrepresented communities. Early detection of lung cancer is highly considered to be a management tool for effective treatment.

The U.S. Preventive Services Task Force (USPSTF) recommends annual screening against lung cancer for individuals between 55 and 80 years of age who have a smoking history of more than 30 packs per 12 months or have ceased smoking for more than 15 years [62]. However, research has also reported that about 25% of lung cancer patients are non-smokers, suggesting that genetics also plays a vital role in the carcinogenesis of lung cancer, as well as pollutants from anthropogenic and natural sources, such as particle matter (PM), gases (benzene, sulfur dioxide, carbon monoxide, radon), organic compounds (polycyclic aromatic hydrocarbons (PAHs), methylmercury), metal (lead, cadmium, chromium, nickel), and ionizing/non-ionizing radiation [63,64,65]. Yin et al. [55] discovered that carriers of the CT and TT genotype of miR-26a-1 rs7372209 and miR-16-1 rs1022960 who have been exposed to cooking oil fumes had a higher risk of developing lung cancer compared to those who were not exposed, with odds ratios (ORs) of 1.743 (95% CI = 1.038–2.753, *p* =  0.036) and 2.326 (95% CI = 1.409–3.843, *p* = 0.001), respectively [55]. They also found out that carriers of CC genotype were more likely to develop lung cancer when exposed to cooking oil fumes (OR = 2.177, 95% CI = 1.274–3.719, *p* = 0.004). The carriers of AG and GG genotype of miR-605 rs2043556 had a 2.194-fold higher chance than AA carriers (OR = 2.194, 95% CI = 1.277–3.767, *p* = 0.004) of developing lung cancer [46]. In a similar study, Zhang et al. [56] investigated the additive interactive effect of three SNPs (rs2608053, rs1561927, rs13254990) and smoking status on lung cancer genesis. They found out that patients carrying AG or GG genotype of rs151927 who were exposed to smoking were 7.18 times at risk of lung cancer compared to those with AA genotype with no smoking exposure (OR = 7.108, 95% CI = 2.36–21.37) [56]. Patients identified with both AG/GG genotypes of rs2608053 and rs 13254990 who were smokers were 5.89 and 5.47 times more likely to manifest lung cancer compared to GG genotype carriers and non-smokers, respectively [56].

Hosgood et al. [66] examined the gene and household air pollutant (HAP) interactions associated with lung cancer among non-smokers. They found out that the risk of lung cancer increased 20% and 30% among those using HAP of solid fuel (OR = 1.2, 95% CI = 1.0–1.4) and coal (OR = 1.3, 95% CI = 1.0–1.6), respectively. They also observed that individuals with SNP rs9387478 located on chromosome 6 of the gene GOPC had a *p*-value interaction to solid fuel equal to 0.05 and SNP rs4488809 and rs2395185 located on chromosome 6, 3 had a *p*-value interaction to coal use equal to less than 0.05, demonstrating evidence of a statistically significant gene–HAP interaction. Another study evaluating the association between gene–radon interaction and lung cancer among uranium miners noted that the chromosome 5q23.2 with SNP rs6891344 and rs11747272 had an OR interaction effect estimated at 3.9 and 3.4, suggesting that susceptibility to lung cancer was higher among uranium workers exposed to radioactive gas radon [67]

### 3.3. Gene–-Environment Interactions in the Development of Colorectal Cancer

Colorectal cancer (CRC) is a heterogeneous disease that initiates through the continuous accumulation of hereditary and epigenetic changes, leading to alteration of normal colon mucosa into benign neoplasm, and subsequently into invasive carcinoma [68]. In the US, the lifetime risk for CRC is about 1 in 15 for men and 1 in 19 for women. CRC is classified as the third leading cause of deaths after lung and prostate cancers in men and breast cancer in women [3]. It has been estimated that 147,950 new cases and 53,200 deaths from CRC will occur in 2020 [3]. The incidence and mortality rates of CRC vary widely by geographical locations across the globe. Approximately 55% of CRC cases occur in industrialized countries. However, the highest mortality is observed in developing countries [69,70]. The poor survival rate in developing countries may be attributed to the absence of adequate health care services, under-diagnosis, and economic conditions [53]. However, the molecular mechanisms influencing the high susceptibility to CRC are still under investigation.

Scientists have suggested that gene instability and environmental factors are predominant factors responsible for CRC [71,72]. Gene instability involves structural aberrations or changes in chromosome copy number, leading to loss of functionality in tumor suppressor genes like *APC*, *P53*, *PI3K,* and *WNT*, and this accounts for about 85% of CRC cases [72]. Aberrant DNA methylation in the mismatch repair system (MMR) could bring about an increase in the mutation rate by more than 100-fold compared to normal cells [73]. The immediate consequences are the powerlessness of the genome to faithfully replicate [34,74], and inactivation of genes responsible for MMR, such as *MLH1* and *MSH2*, accounting for between 2–3% of CRC cases [75]. For instance, a twin study from Finland, Denmark, and Sweden found out that 35% of CRC cases were hereditary [76].

On the other hand, Andersen et al. [77] noticed that alcohol, red and processed meat, and dairy product consumption, as well as lack of physical activities and high BMI, significantly increased the risk of CRC after adjustment of other covariates like age and sex [77,78]. From their genome-wide association studies (GWAS), Song et al. [57] discovered that individuals consuming high levels of alcohol were 2.47 times more likely to develop CRC compared to those who never took alcohol [57]. They also noticed individuals with SNP rs6687758 close to the *DUSP10* gene had 1.47 odds ratio greater compared to non-alcohol drinkers with CRC. Their research also suggested that SNP rs4444235 at 14q22.2 and SNP rs2423279 at 20p12.3 may interact with regular exercise and aspirin use in CRC risk [57]. Other investigators have also noted that alcohol induces carcinogenesis by altering alcohol-metabolizing enzymes, including alcohol dehydrogenase (ADH), acetaldehyde dehydrogenase (ALDH), and cytochrome P450 2E1 (*CYP2E1*), and by modulating the activity of the methylenetetrahydrofolate reductase (MTHFR) enzyme, which plays a crucial role in folate metabolism [79].

### 3.4. Gene–Environment Interactions in the Development of Bladder Cancer

Bladder cancer is ranked as the fourth most common cancer worldwide and causes more deaths in men compared to women [3]. The WHO estimated that more than 81,000 new cases of bladder cancer will occur in 2020 and 17,900 deaths are predicted from the disease in 2020 [3]. Bladder cancer is also considered to be a smoking-related disease. Increased awareness and understanding of its epidemiology and related risk factors could help in developing and implementing preventive measures to reduce bladder cancer incidence [80,81]. Koutros et al. [82] pointed out that the leading cause of bladder cancer risk is exposure to occupational pesticides [82]. Lesseur et al. [83] conducted a population-based control study in New Hampshire to evaluate the gene–environment interactions, focusing on genetic polymorphism and low-dose exposure to arsenic [83]. They pointed out that susceptibility to bladder cancer may be related to the variation in a gene involved in arsenic metabolism and oxidative stress [83]. They also found that individuals with AQP3 genotype had an odds ratio (OR) two times greater for bladder cancer in the high arsenic exposure group (OR = 2.2, 95% CI = 0.8–6.1) compared to those in the low arsenic exposure group with the same genotype (OR = 0.8, 95% CI = 0.6–1.1 [83]. From further analysis of the GST variants, they demonstrated that individuals with elevated arsenic exposure had an OR of 5.4 (95% CI = 1.5–20.2), whereas those with low arsenic exposure has an OR of 0.8 (95% CI = 0.6–1.2) [83]. Similarly, another study investigating the association between genetic polymorphisms of MOP, COMT, MnSOD, and NQO1 interactions with environmental factors including coffee, smoking, exposure to aromatic amines (AA), and polycyclic aromatic hydrocarbons (PAHs) discovered that MnSOD Val/A\Val genotype positively increased the risk of bladder cancer, showing an OR equal to 1.91 (95% CI = 0.12–0.80) [84]. The risk of bladder cancer effect was more amplified when this genotype was combined with smoking (OR = 7.32) and PAH (OR = 3.02) [84]. Other studies evaluating the gene–environment interactions related to genetic polymorphisms and caffeine metabolism showed no evidence of interaction between *CYP1A1, CYP1A2*, and *NAT2* and consumption of caffeine in bladder cancer risk, but a statistically significant effect was observed when adjustments were not made in multiple comparisons [85,86].

### 3.5. Gene–Environment Interactions in the Development of Breast Cancer and Ovarian Cancer

Breast and ovarian cancers are considered female hormone-related cancers and collectively share behaviors that are affected directly and indirectly to xenoestrogen exposure [87]. The association between environmental and lifestyle risk factors may be stronger or weaker for women with greater genetic risk of breast or ovarian cancer compared to women with smaller genetic risk [88]. Therefore, gene–environment interaction studies will help to identify the women at high risk of breast and ovarian cancers and will be necessary for recognizing the new genetic risk and better understanding the familiar factors [89]. Research in that area will also make it possible to give additional screening and preventive measures to the women at high risk [90].

Breast and ovarian cancers were the most commonly diagnosed types of cancer in women in 2019. They both accounted for more than 293,800 new cases and 55,730 deaths, which correspond to about 17% and 9% of all new cancers diagnoses and deaths in the US, respectively [3]. Genetic testing and counseling studies found that women carrying *BRCA1* and *BRCA2* in their family lineage were at 72% and 69% cumulative risk of breast cancer by the age of 80, respectively, and were at 41% (in *BRCA1*) and 17% (in *BCRA2*) cumulative risk of developing ovarian cancer by the age of 80 [91,92]. However, the occurrence of breast and ovarian cancers in women may also be dependent on other factors, such as the history of endometriosis, use of menopausal hormones therapy (MHT), use of oral contraceptive, tubal ligation, breastfeeding, and foodborne mutagens [93].

Rudolph et al. [94] conducted a study of G × E interactions toward estrogen-receptor (ER)-positive and ER-negative risk of breast cancer with 41 newly susceptible genes and evaluated the overall breast cancer risk with environmental factors, such as age, breastfeeding, height, BMI, smoking status, and alcohol intake [94]. They found an odds ratio (OR) equal to 1.09 (CI = 1.04–1.15) for G × E interactions risk of ER-negative breast cancer between the rs12422553 located in chromosome 12 and the women with a height greater than 170 cm compared to women that were shorter [94]. A specific interaction was also discovered in women carrying rs941764 located in chromosome 14 and alcohol intake. A stronger interaction was noticed among smokers and chromosome 6 with rs11242675 compared to an odds ratio of 1.13 for non-smokers [94]. Another study conducted by the Breast Cancer Association Consortium also reported strong G × E interactions between CFLAR-rs7558475 and cigarette smoking (OR = 0.77, 95% CI = 0.67–0.88, *p* = 0.00018), and between 5q14-rs7707921 and alcohol consumption (OR = 1.36, 95% CI = 1.16–1.59, *p* = 0.000019) in relation to ER-negative breast cancer risk [95]. Given the complex interactions between hormonal factors, age, weight gain, height, insulin level and growth factors, and genetic susceptibility in women, it would be important to analyze some underlying causes of variations in these interactions.

Qui et al. [88] found that women eating bigger amounts of fried food tend to have an unhealthy dietary pattern, higher calorie intake, lower levels of physical activity, and more significant levels of sedentary habits [88]. Consequently, the intake of fried food might be an indicator of an undesirable eating routine and lifestyle and may also collaborate with a genetic predisposition to obesity and high mortality rate [96]. Hence, it is recommended that women reduce the quantity of fried food consumption because of the genetic predisposition to obesity, which may further initiate cancer development. Also, it has been pointed out that a high intake of fish and long-chain polyunsaturated fatty acids is associated with genetic alteration that may induce a long-term effect of weight gain in women [97].

### 3.6. Gene–Environment Interactions in the Development of Prostate Cancer

Prostate cancer (PC) is a form of cancer that starts in the prostate gland of the male reproductive system and emerges as the result of a mutation in tumor suppressor genes or oncogenes, which deregulates the homeostatic functions and causes the transformation of normal cells into malignant cells [98,99]. PC is considered the leading non-cutaneous cancer or most commonly diagnosed cancer in men worldwide, even though the incidence and mortality rates fluctuate substantially by population and geographic location [100]. The incidence rate of PC tends to be higher in developed countries. In the US, it was estimated that about 174,650 men were diagnosed with prostate cancer in 2019 and 31,620 of those diagnosed would die from prostate cancer [3]. The mortality rate of PC seems to be higher in developing countries compared to industrialized countries [4]. Men of African descent, including afro-Caribbean, sub-Saharan African, and African American men, suffer the highest mortality rate from PC compared to other ethnicities; while the death rate ranges from 18.7 to 29.0 per 100,000 in the general population [1,100], the death rate is even higher in African American men, at 43 per 100,000 people, which accounts for about 60% of the prostate cancer death rate compared to other racial groups [101].

Established etiological variables responsible for PC development include increased age, family history, Black ethnic background, and genetic factors. Lifestyle choices and some environmental factors are modifiable. Cigarettes, for example, contain more than 7000 hazardous chemicals listed as cancer-causing and inflammatory agents in humans [85]. Furthermore, concerning behavioral factors, factors such as lack of physical activity, heavy alcohol consumption, and high intake of caffeine may contribute to several disorders such as metabolic syndrome, insulin resistance, alteration of hormone levels, immune system deficiencies, inflammation, and other disorders [102]. For instance, Neslund-Dudas et al. [58] investigated G × E interaction in rs10486567 CC genotype among African American men exposed to lead and cadmium and discovered that men carrying the CC genotype had a 2.03 risk of prostate cancer (CI = 0.99–4.19, *p* = 0.05) after living in a household that was built after 1950 compared to controls, and had stronger G × E interactions with occupational lead exposure (OR = 2.62, 95% CI = 1.03–6.68, *p* = 0.04) [58]. Another G × E interactions study demonstrated that high caffeine intake may increase the risk of prostate cancer [103]. The key hereditary loci that affect the digestion of caffeine are cytochrome P450 (*CYP1A1; CYP1A2*) and the aryl hydrocarbon receptor (AHR) [104]. The role of *CYP1A2* enzymes is to metabolize caffeine; meanwhile, the *ARH* regulates the transcription of the *CYP1A2* [105]. Consequently, the interaction of these variants with various allelic genes may increase the risk for PC [102]. However, further investigations are needed to verify if caffeine decreases PC risk through interactions with other chemical substances. There may be a backward connection between the amount of caffeine intake expanding alleles and the outcomes [102].

### 3.7. Challenges in Gene–Environment Interactions in the Cancer Intervention and Prevention

The most significant challenge in studies of G × E interactions is the multifaceted nature of the environmental variables [106]. This complex situation has raised concerns over the validity of the results generated from many scientific studies on G × E interactions [107,108], primarily because environmental factors are heterogeneous in various aspects. Secondly, the modalities of measurement at different places, such as the home, community, and workplace, can vary enormously, leading to errors in estimates and the power to detect the effects [107]. Also, human exposures and biological impacts can fluctuate impressively from the period of conception through adulthood. Hence, G × E studies have the potential to induce a non-linear association because the impacts of environmental factors on biological systems are pleiotropic in nature and likely to induce overlapping effects on malignant growth [109,110]. Since exposures are often spatially, temporally, and socially dependent, investigators conducting research on G × E interactions agree that exposure effects and disease outcomes differ across genotypes and geographic settings. Observed differences may be related to many factors, including small sample sizes, which lower the power of testing G × E interactions; ambiguity in measuring environmental exposures; difficulty in incorporating in measurement terms the amount of environmental exposure; and/or ranges of gene and environmental variances [93]. Consequently, numerous environmental exposures can be profoundly associated with each other, making it hard to distinguish the impact of a single exposure [111].

The research methodology and statistical analysis of G × E studies have also encountered challenges. In small-scale investigations, statistical programming such as SAS, STATA, and SPSS (SAS Institute Inc., Cary, NC, USA) can be used for G × E analyses [112]. However, these programs cannot be used to effectively manage large sample sizes with high data volumes from genome-wide association studies (GWAS) or more complex models. Such large studies on G × E interactions will require the use of a more robust programming system [108,113]. Therefore, it is necessary to develop novel programming systems that are able to manage larger amounts of data.

## 4. Conclusions

Cancer remains a significant public health concern worldwide and is the second leading cause of death in the United States. This review highlights the prominent role of gene–environment (G × E) interactions in the development of lung, colon, bladder, breast, ovary, and prostate cancers. Scientific evidence from studies on G × E interactions has revealed that the epidemiological profile of cancer etiology is complex and multifactorial. Case-control and follow-up studies have demonstrated that cancer can be initiated from either hereditary factors alone, environmental factors alone, or a collaboration between genetics and environment. It has also been reported that genetic variables including alleles, chromosome location or number, SNPs, and RS number may interact with environmental factors such as exposure to arsenic, benzene, polychlorinated biphenyl (PCB), polycyclic aromatic hydrocarbons (PAHs), chlorinated dioxin, etc., and modifying factors such as poor diet, smoking, physical activity, and other lifestyle factors to affect the cancer risk in human populations. Nevertheless, the specific mechanisms by which the interaction of environmental and genetic variables influences the risk of cancer remain unclear. Because some genes can only be activated or downregulated under specific environmental conditions, the health outcomes from exposure to environmental risk factors vary depending on individual hereditary. Also, it is often difficult to accurately measure and quantify the role played by environmental factors from G × E interactions studies, even though the assessment of genetic effects may be easier to evaluate. Environmental exposure takes into consideration factors such as chemical concentration, duration of exposure, geographical location, and individual or cultural behaviors. These heterogeneous variables are likely to confound, produce biases, and probably create a lack of data replication in both observational and randomized clinical studies of G × E interactions. The determination of individuals or populations at risk of developing cancer requires a thorough understanding of the role played by genes and environmental factors, and, most importantly, the interplay of G × E interactions in the initiation, promotion, and progression of cancer. Recently, it has been highly recommended for researchers to increase their population sample sizes, incorporate functional annotations, and utilize improved statistical tools and computational models in order to produce more robust and dependable results and thereby increase the scientific strength of G × E interactions studies. Hence, there is a critical need to further develop and apply novel epidemiological approaches, analytical tools, and computational models to investigate and understand the underlying mechanisms of G × E interactions and their prominent role in the initiation, promotion, and progression of human cancers.

## Figures and Tables

**Table 1:** Major findings on gene–environment interactions associated with human cancers.

Cancers Site/Types of Study	Genes (SNPs, Genotype, RS-Number, Chromosome, Allele) of Interest	Environmental Exposures	Key Findings on Gene–Environment Interactions Based on OR, RR, and/or HR
Lung cancer (LC) case-control studies [53]	miR-26a-rs7372209 (TT, CT, AA)	Regular exposure to cooking oil fume	TT and CT genotype carriers of miR-26a-1 rs7372209 and miR-16-1 rs1022960 who had been exposed to cooking oil fumes had a higher risk of developing LC compared to those who were not exposed to cooking oil fumes (OR = 1.743, 95% CI = 1.038–2.753, *p* = 0.036) and (OR = 2.326, 95% CI = 1.409–3.843, *p* =0.001), respectively.
	miR-605 rs2043556		
	miR-16-1 rs1022960		
Lung cancer (LC) hospital-based case-control studies [54]	rs2608053 (AA, GG, AA/GG)	Heavy cigarette smoking	AG or GG genotype of rs151927 smoking were 7.18 times at risk of LC compared to those with AA genotype with no smoking exposure (OR = 7.108, 95% CI = 2.36–21.37). AG/GG genotypes of rs2608053 and rs13254990 polymorphism were more likely to develop lung cancer compared to non-smokers.
	rs1561927 (AA, GG, AA/GG)		
	rs13254990 (AA, GG, AA/GG)		
Colon cancer case-only and case-control studies from GWAS [55]	rs10849432 at 12p13.31	Alcohol consumption, lack of physical activity, use of aspirin, high intake of red and processed meat	SNP rs4444235 at 14q22.2 and regular exercise and the SNP rs2423279 at 20p12.3 and regular aspirin use may increase the risk of colorectal cancer initiation.
	rs11196172 at 10q25.2		
	rs4444235 at 14q22.2		
	rs2423279 at 20p12.3		
	rs2241714 at 19q13.2		
	rs1957636 at 14q22.3		
Bladder cancer case-control studies [56]	rs1695 AA;	Environmental exposure to arsenic	Genotype–arsenic interactions in the high exposure group were linked to increased risk of bladder cancer initiation.
	GSTP1 AG		
	Ile105Va GG		
Breast (BC) case-control studies [57]	rs4808801 located on chromosome 19	Age at first birth and menarche, number of pregnancies, obesity, smoking, and heavy alcohol drinking	Strong association was found between BC risk, rs4808801, and four and more full-term pregnancies (OR = 5.08, 95% CI = 0.77–0.93, *p* = 2.0 × 10^−4^). Interactions were also found between smoking, rs 11242675, and BC risk (OR = 1.13, 95% CI = 1.06–1.21, *p* = 3.4 × 10^−4^), and also between alcohol consumption, BC risk, and rs941764.
	rs11242675 located on chromosome 6		
	rs941764 located in chromosome 14		
	rs12422552 located in chromosome 12		
	rs16857609 located in chromosome 2		
Prostate cancer case-only [58]	CC, TT, CT of JAZF1 genotype	Environmental exposure to lead and cadmium, and heavy cigarette smoking	Strong G × E interactions with carrier of CC genotype, exposure to Pb in houses built before 1960, and PC risk compared to CT or TT genotype (OR = 1.81, 95% CI = 1.04–3.16; *p* = 0.036).

OR: odds ratio; RR: relative risk; HR: hazard risk; CI: confidence intervals; MHT: menopausal hormone therapy; BMI: body mass index; GWAS: genome-wide association studies.
